# Reinforcing Stromal Cell Spheroid Through Red‐Light Preconditioning for Advanced Vascularization

**DOI:** 10.1002/advs.202500788

**Published:** 2025-04-25

**Authors:** Yu‐Jin Kim, Hyeok Kim, Dong‐Hyun Lee, Yeong Hwan Kim, Jae‐Hyun Park, Woo‐Sup Sim, Jin‐Ju Kim, Kiwon Ban, Soong Ho Um, Hyun Ji Park, Michael E. Davis, Hun‐Jun Park, Suk Ho Bhang

**Affiliations:** ^1^ School of Chemical Engineering Sungkyunkwan University Suwon 16419 Republic of Korea; ^2^ Center for Biomaterials Biomedical Research Institute Korea Institute of Science and Technology Seoul 02792 Republic of Korea; ^3^ Department of Biomedicine & Health Sciences College of Medicine The Catholic University of Korea Seoul 06591 Republic of Korea; ^4^ Department of Biomedical Sciences City University of Hong Kong Hong Kong 999077 China; ^5^ Department of Molecular Science and Technology Ajou University Suwon 16499 Republic of Korea; ^6^ Wallace H. Coulter Department of Biomedical Engineering Georgia Institute of Technology & Emory University Atlanta GA 30332 USA; ^7^ Children's Heart Research & Outcomes (HeRO) Center Children's Healthcare of Atlanta & Emory University Atlanta GA 30322 USA; ^8^ Division of Cardiology Department of Internal Medicine Uijeongbu St. Mary's Hospital The Catholic University of Korea Seoul 06591 Republic of Korea; ^9^ Cell Death Disease Research Center College of Medicine The Catholic University of Korea Seoul 06591 Republic of Korea

**Keywords:** angiogenesis, arteriogenesis, myocardial infarction, OPBM‐preconditioning, stromal cell spheroid

## Abstract

Despite the promising potential of stromal cell therapy in treating myocardial infarction (MI), its effectiveness is limited by poor cell retention and engraftment in ischemic environments. This study introduces a novel strategy that combines the preconditioning of human adipose‐derived stromal cells (hADSCs) using OLED‐based photobiomodulation (OPBM) and culturing these cells into 3D spheroids. The preconditioned 3D spheroids (APCS group) exhibit significantly enhanced angiogenic, arterialized, and tissue remodeling capabilities compared with those of traditional 2D cultures and non‐preconditioned spheroids. In vivo transplantation of these spheroids into the border zone of infarcted area significantly improve cardiac function and reduce adverse remodeling by enhancing anti‐fibrosis and angiogenesis including arterialization. The combined strategy with OPBM preconditioning and 3D spheroid culture system can enhance therapeutic potential of hADSCs with multiple paracrine effects for cardiac repair. This novel approach provides next generation of cell therapeutics to overcome the limitation of adult stromal cell therapy in patients with post‐MI heart failure.

## Graphical abstract

This study presents a novel approach to enhance stromal cell therapy for myocardial infarction by combining OLED‐based photobiomodulation (OPBM) preconditioning with 3D spheroid culture of hADSCs. The engineered spheroids improve angiogenesis, arterialization, and cardiac function in vivo, offering a next‐generation therapeutic strategy to overcome poor cell retention and efficacy in ischemic environments.

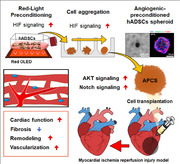

## 1 Introduction

Myocardial infarction (MI) is a critical health concern with ≈20% mortality rate within the first year of onset.^[^ ^1^ ^]^ MI primarily results from impaired supply of oxygen and nutrients to the myocardium, which necessitates the prompt restoration of blood flow to mitigate the damage or myocardial damage.^[^ ^2^ ^]^ Among the diverse strategies implemented for managing MI, stromal cell transplantation shows considerable promise because stromal cells promote angiogenesis and exert immunomodulatory and anti‐fibrotic properties, all of which are essential for myocardial repair.^[^ ^3^ ^]^ However, despite the potential of improving clinical outcomes with the use of stromal cells for the treatment of ischemic heart disease, many clinical trials frequently fall short of expectations, primarily because of the challenges associated with cell survival and engraftment in ischemic environments.^[^ ^4^ ^]^ Currently, to overcome the limited therapeutic effects of adult stromal cells for cardiac repair, we need to develop novel advanced strategies to increase not only cell retention and engraftment, but also functional revascularization with perfusable vascular networks in infarcted hearts.

Recent studies have shown that aggregating stromal cells into in 3D structures such as spheroids, significantly improves engraftment, possibly due to the innate ability of spheroids to internally create a hypoxic environment that substantially amplifies the angiogenic capacity of stromal cells.^[^ ^5^ ^]^ Adopting this approach for the treatment of ischemic diseases has significantly boosted therapeutic outcomes.^[^ ^5^, ^6^ ^]^ Despite the promising potential of spheroid‐based stromal cell therapies for MI, the amplification of their therapeutic efficacy remains a significant but underexplored area of regenerative medicine. Various methods have been proposed for injecting biomaterials or growth factors along with spheroids to tackle this challenge. However, the introduction of exogenous materials poses problems in terms of biocompatibility and clinical applications.^[^ ^7^ ^]^ In the other hand, preconditioning techniques were explored to enhance de novo stromal cell functions in 3D cell cultures. Stromal cells exposed to a hypoxia before being subjected to 3D culture exhibited higher HIF‐1α levels, which could enhance the secretion of angiogenic factors and promoted bone regeneration in vivo.^[^ ^8^ ^]^ Despite this, translation is difficult as specialized equipment is required to maintain a hypoxic environment.^[^ ^9^ ^]^ As an alternative to hypoxia, we previously demonstrated that exposing human adipose‐derived stromal cells (hADSCs) to red light through organic light‐emitting diodes (OLEDs) over time enhanced their angiogenesis capability.^[^ ^10^ ^]^ Photobiomodulation (PBM), particularly in the red light range (600–700 nm), is known to modulate cellular activity by targeting cytochrome c oxidase (CCO), a key enzyme in complex IV of the mitochondrial electron transport chain.^[^ ^11^ ^]^ Photon absorption by CCO alters its redox state, leading to a transient increase in mitochondrial membrane potential and the generation of reactive oxygen species (ROS).^[^ ^12^ ^]^ These ROS act as secondary messengers that regulate various redox‐sensitive signaling pathways, thereby influencing cellular functions such as proliferation, survival, and angiogenesis. Notably, in our previous study using red OLEDs, this effect was not associated with nitric oxide release or ATP synthesis, but was primarily mediated by increased intracellular ROS, generated through red OLED‐induced stimulation of mitochondrial CCO and the activation of the receptor tyrosine kinase (RTK) pathway. These signals collectively led to the upregulation of VEGF and HGF expression via HIF‐1α signaling.^[^ ^10^ ^]^ Furthermore, OLED‐induced ROS elevation transiently increased expression of heat shock proteins (e.g., HSP27 and HSP90α), contributing to cellular protection and enhanced stem cell functions.^[^ ^10^ ^]^ Moreover, during cell detachment with trypsin and reattachment, the attachment ability was improved and gene expression of angiogenic factors continued to increase.^[^ ^10^ ^]^

Furthermore, beyond angiogenesis, collateral artery formation is required for functional revascularization after MI.^[^ ^13^ ^]^ This usually occurs in injured hearts primarily through arteriogenesis, which involves the creation of blood vessels from existing arteries by the occluded artery's pressure gradient.^[^ ^13^, ^14^ ^]^ However, in the view point of vascular regeneration using stromal cells therapy, rapid arterialization of sprouting capillaries and connection with host vascular networks are quite important factors to create functional revascularization. There are some reports that preconditioning the cells before transplantation into the ischemic region increases their arterialized ability, which improves therapeutic efficiency in ischemic diseases by enhancing collateral artery formation.^[^ ^15^ ^]^ For example, blood flow was increased in the hindlimb by improving arterialization in the ischemic region by transplanting immune cells with enhanced HIF‐1α expression after their exposure to a hypoxic environment.^[^ ^15^ ^]^ Additionally, stromal cells induced arterialization in ischemic regions through the paracrine effect.^[^ ^15^, ^16^ ^]^ Arterialization was improved in myocardial ischemia by injecting stromal cells with enhanced ANGPT‐1 expression after genetic modification.^[^ ^15^ ^]^ Thus, the preconditions applied to cells to enhance arterialization ability are associated with intracellular HIF‐1α signaling and angiogenic paracrine factor secretion. Notably, these modifications are naturally observed when stromal cells are cultured in 3D because of increased internal hypoxic environment and enhanced cell—to–cell interaction.^[^ ^17^ ^]^ Therefore, we hypothesize that spheroid‐based stromal cell transplantation may offer significant advantages for enhancing arterialization in ischemic regions.

**Figure 1:**
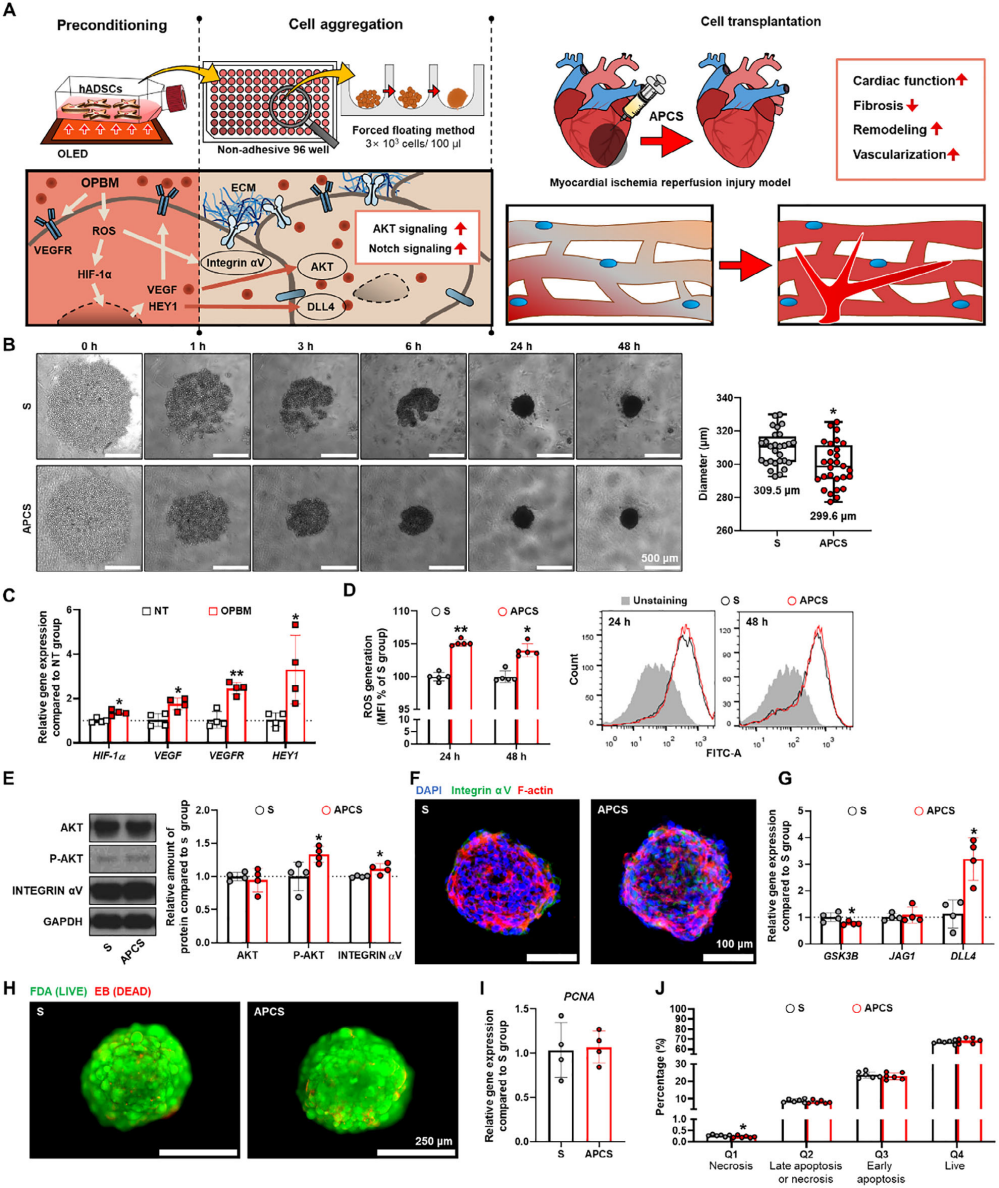
Morphology, cellular properties, and viability of reinforced human adipose‐derived stromal cells (hADSC) spheroids. A) Schematic describing the preparation of hADSC spheroids after preconditioning with red light irradiation. B) Representative morphology of hADSC spheroids over time; diameter of spheroids measured at 48 h after aggregation (scale bar: 500 µm, ^*^ *p* < 0.05 vs S group). C) Relative expression of hypoxia‐related genes in hADSCs after preconditioning with red light irradiation. The no treatment (NT) group was used as the control (*n* = 4, ^*^ *p* < 0.05 compared with the NT group). D) Staining of intracellular ROS using dichloro‐dihydro‐fluorescein diacetate (DCF–DA) and its quantification expressed as percentage of the median fluorescence intensity (MFI). The S group was used as the control (*n* = 5, ^*^ *p* < 0.05 vs S group). E) WB analyses of Integrin αV, phospho‐AKT, and AKT levels in reinforced hADSC spheroid. GAPDH was used as the loading control (*n* = 4, ^*^ *p* < 0.05 vs S group). F) Representative images of Integrin αV and F‐actin staining in the reinforced hADSC spheroids (Integrin αV: green, F‐actin: red, and nuclei: blue, scale bar: 100 µm). G) Relative expression of *GSK3B*, *JAG1*, and *DLL4* in reinforced hADSC spheroids was analyzed using qRT–PCR. The S group was used as the control (*n* = 4, ^*^ *p* < 0.05 vs S group). The cellular viability of hADSCs was evaluated using H) the fluorescein diacetate (FDA)‐ethidium bromide (EB) assay (live cells stain, green; dead cells stain, red; scale bar, 250 µm), I) expression of *PCNA*, and J) fluorescence‐activated cell sorting (FACS) analysis of apoptosis in 2D, S, and APCS groups stained with Annexin V/7‐AAD (*n* = 6, ^*^ *p* < 0.05 vs S group).

In this study, we aimed to improve the angiogenic and arterialized capabilities of stromal cells by integrating preconditioning technique with 3D culture method. This dual strategy was designed to increase the efficiency of stromal cell transplantation in MI therapy and is illustrated in **Figure** **1**A. hADSCs were preconditioned in 2D form using light before aggregation into spheroids, which were transplanted into a myocardial ischemia‐reperfusion injury model to evaluate their therapeutic efficacy. Therefore, we aimed to enhance the angiogenic capability of spheroids without the introduction of exogenous substances or specialized equipment by applying the OLED‐based photobiomodulation (OPBM) technique to precondition 2D cultured hADSCs before culturing them in 3D. By using this innovative approach, we aimed to establish a novel paradigm for MI treatment and potentially revolutionize the clinical applicability and success rate of MSC‐based therapies.

## 2 Results

### 2.1 Angiogenic‐Preconditioned hADSCs Spheroid Characterization and Viability

We investigated whether spheroids composed of OPBM‐preconditioned hADSCs (angiogenesis‐preconditioned spheroid (APCS) group) were more suitable for the treatment of ischemic diseases than the cells that were not preconditioned (S group) without damaging cell viability (Figure 1). Figure 1A presents an outline the spheroid preparation process using hADSCs. Preconditioned was performed using red light for myocardial infarction therapy. The APCS group formed cell aggregates more rapidly than the cells in the S group (Figure 1B). After 48 h, the APCS group showed an average diameter of 299.3 ± 13.4 µm, which was smaller than that of the S group cells (309.5 ± 10.4 µm). *HIF‐1α*, *VEGF*, *VEGFR*, and *HEY1* gene expressions in the cells were evaluated immediately after exposure to light (Figure 1C). In conformance with the results of our previous study, OPBM treatment improved the angiogenic efficiency of hADSCs with respect to angiogenic preconditioning.^[^ ^10^ ^]^ Additionally, OPBM treatment increased *HIF‐1α* expression; consequently, HIF‐1α signaling induced upregulation of the representative angiogenic factors such as VEGF and HEY1.^[^ ^18^ ^]^ OPBM treatment increased VEGF‐induced upregulation of VEGFR expression.^[^ ^19^ ^]^ Next, the specificity of spheroids produced from the angiogenic‐preconditioned hADSCs (APCS group) was investigated and compared with that of the S group cells. Intracellular ROS concentration in the cells constituting the spheroids was measured after 24  and 48 h of cell aggregation. Intracellular ROS concentration was higher in the APCS group than in the S group (Figure 1D). Western blotting (WB) analysis confirmed that p‐AKT and integrin αV expression was higher in the APCS group than in the S group (Figure 1E). Integrin αV has been shown to promote migration and invasion through the FAK/STAT3/AKT signaling pathway.^[^ ^20^ ^]^ Additionally, integrin αV was expressed both inside and outside the spheroid in the APCS group (Figure 1F). The uniform expression of integrin αV throughout the spheroid suggests enhanced cell—to–cell and cell–to–ECM interaction, which may contribute to the stable activation of the AKT signaling pathway.^[^ ^21^ ^]^ *GSK3B* expression significantly decreased in the APCS group compared with that of the S group (Figure 1G). Investigating the expression of representative Notch ligands in spheroids showed that *DLL4* expression was higher in the APCS group than in the S group (Figure 1G).^[^ ^22^ ^]^ The increased *HEY1* expression observed immediately after preconditioning may have stimulated Notch signaling in hADSCs.^[^ ^23^ ^]^ We examined the viability of light‐precondition hADSCs after aggregation (48 h) through FDA/EB fluorescence staining results, and no significant difference was observed between the groups (Figure 1H). The expression of the proliferation‐associated gene *PCNA* did not differ between the APCS and S groups (Figure 1I). In contrast, the APCS group showed a slightly lower population of necrotic cells than did the S group (Figure 1J). TUNEL staining was also performed to assess apoptotic activity within the spheroids. Representative images (Figure S1, Supporting Information) illustrate a reduction in apoptotic cells, visualized as green fluorescence, in the APCS group compared to the S group. Meanwhile, intact nuclei, stained with DAPI (blue), remained consistent across all conditions. In conclusion, we confirmed that hADSC preconditioning using light did not affect cellular viability but significantly enhanced the angiogenic capacity of the hADSC spheroids.

### 2.2 Effects of Light‐Preconditioning on Hypoxia‐Related Signaling in hADSC Spheroids

**Figure 2:**
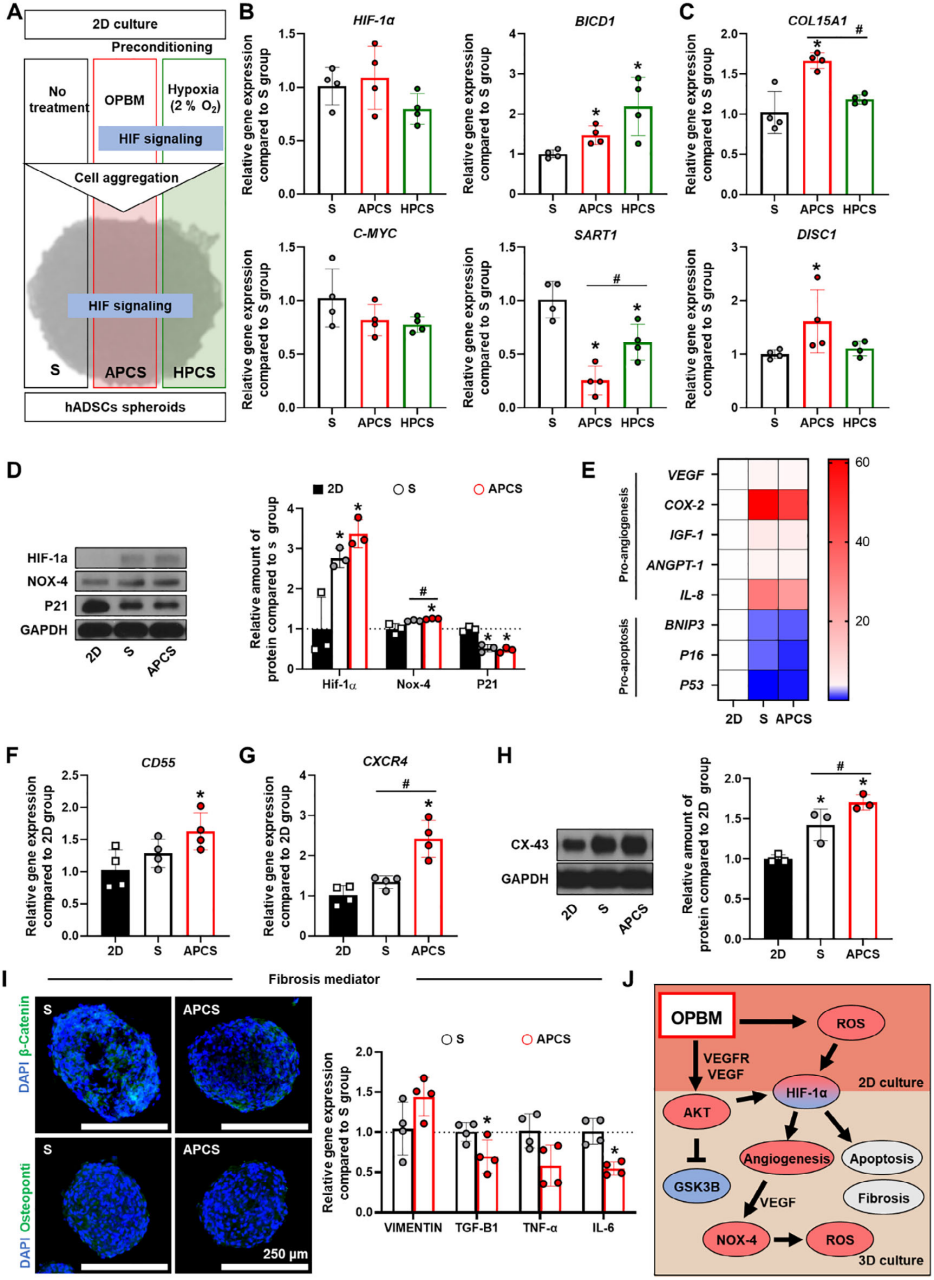
3D culture strengthened and maintained the effect of preconditioning. A) Scheme figure of reinforcing hADSC spheroids. B) Relative gene expression of HIF‐1α, BICD1, c‐MYC, and SART1 in hADSC spheroids analyzed using the S group as the control. (*n* = 4, ^*^ *p* < 0.05 vs S group). C) Relative gene expression of COL15A1 and DISC1 in hADSC spheroids analyzed using the S group as the control (*n* = 4, ^*^ *p* < 0.05 vs S group, ^#^ *p* < 0.05 vs each group). D) Western blotting results of HIF‐1α, NOX‐4, and P21 in hADSCs. GAPDH was used as the loading control (*n* = 3, ^*^ *p* < 0.05 vs 2D group, ^#^ *p* < 0.05 vs each group). E) Relative gene expression of angiogenesis and apoptosis related genes in hADSCs analyzed using the 2D group as the control (*n* = 4). Relative gene expression of F) CD55 and G) CXCR4 in hADSCs analyzed using the 2D group as the control (*n* = 4, ^*^ *p* < 0.05 vs 2D group, ^#^ *p* < 0.05 vs each group). H) Western blotting results of CX43 in hADSCs. GAPDH was used as the loading control (*n* = 3, ^*^ *p* < 0.05 vs 2D group, ^#^ *p* < 0.05 vs each group). I) Immunostaining for β‐catenin^+^ or Osteopontin^+^ (green) with DAPI (blue) in S and APCS groups; scale bar = 250 µm. Relative expression of *VIMENTIN*, *TGF‐β1*, *TNF‐α*, and *IL‐6* in hADSC spheroids analyzed using the S group as the control. (^*^ *p* < 0.05 vs S group). J) Scheme figure introducing the molecular mechanism in the APCS group.

Culturing cells in a spheroidal structure creates a hypoxic environment in the core of the spheroid,^[^ ^5^, ^24^ ^]^ which activates hypoxia‐related signaling in the cells located at the core. The spheroid‐cultured cells exhibit different characteristics than those of 2D cultured cells.^[^ ^5^, ^6^, ^24^ ^]^ To determine whether APCS preconditioning enhanced cell function greater than hypoxia preconditioned, we compared it with an hypoxia preconditioning spheroid (HPCS) group that was exposed to a hypoxic environment before the induction of cell aggregation (**Figure** **2**A–C). *HIF‐1α* mRNA expression did not differ between of S, APCS, and HPCS groups. *BICD1* plays a role in transporting HIF‐1α to the nucleus^[^ ^25^ ^]^; its expression was higher in the APCS and HPCS groups than in the S group. c‐MYC is a transcription factor that is vital in hypoxia regulation and angiogenesis, and c‐MYC expression did not differ between the groups.^[^ ^26^ ^]^ However, the expression of *SART1*, which interferes with hypoxia‐induced VEGF expression, was significantly reduced in the APCS group that in the S and HPCS groups.^[^ ^27^ ^]^ Additionally, *COL15A1* expression, which increases as a result of HIF‐1α signaling,^[^ ^28^ ^]^ and *DICS1* expression, which supports the homeostatic maintenance of translation during oxidative stress,^[^ ^29^ ^]^ increased in the APCS group than in the other groups (Figure 2C). HIF‐1α signaling‐regulated factors were analyzed to determine whether the APCS group cells improved ischemic disease treatment in terms of angiogenesis relative to currently existing cell therapies. WB analysis of the expression of hypoxia‐related factors showed that HIF‐1α protein expression increased in both the S and APCS groups as compared with the 2D group (Figure 2D). Although no significant difference was observed between S and APCS group, NOX‐4 expression significantly higher in the APCS group than in the other groups. In contrast, the expression of the senescence factor P21 decreased in the S and APCS groups compared with that in the 2D group.^[^ ^30^ ^]^ Next, we analyzed the expression of ischemic disease‐associated HIF‐1α signaling‐regulated factors *VEGF*, *COX‐2*, *IGF‐1*, *ANGPT‐1*, and *IL‐8*, all involved in angiogenesis, and found expression of these genes was significantly enhanced in the S and APCS groups than that observed in the 2D group. Conversely, overexpression of HIF‐1α signaling reduces the therapeutic effect of transplanted cells by inducing apoptosis or fibrosis.^[^ ^31^ ^]^ We found expression of apoptosis‐related genes *BNIP3*, *P16*, and *P53* decreased in the S and APCS groups compared with that in the 2D group (Figure 2E). Particularly, *P16* expression was lower in the APCS group than that in the S group. *CD55* promotes ischemic disease treatment,^[^ ^32^ ^]^ and its expression was higher in the APCS group than in the other groups (Figure 2F). *CXCR4* expression significantly increased in the APCS group than in the other groups (Figure 2G). CXCR4 expression is regulated by HIF‐1α signaling and contributes to cell migration and angiogenesis.^[^ ^33^ ^]^ Additionally, CX43 expression increased in the APCS group (Figure 2H). This result conforms to previous studies showing that hypoxic preconditioning reduced CX43 degradation in cells.^[^ ^34^ ^]^ Furthermore, the expression of fibrosis mediators such as β‐catenin and osteopontin was lower in the APCS group than in the S group (Figure 2I).^[^ ^35^ ^]^ Similarly, the expression of fibrosis‐triggering genes *VIMENTIN*, *TGF‐β1*, *TNF‐α*, and *IL‐6* in the APCS group decreased or remained the same as that in the S group (Figure 2I).^[^ ^36^ ^]^ Figure 2J shows the schematic of HIF‐1α signaling in spheroid containing light‐preconditioned (OPBM) hADSCs.

### 2.3 Improved Vascularization Efficacy of Spheroid After Light‐Preconditioning (APCS)

**Figure 3:**
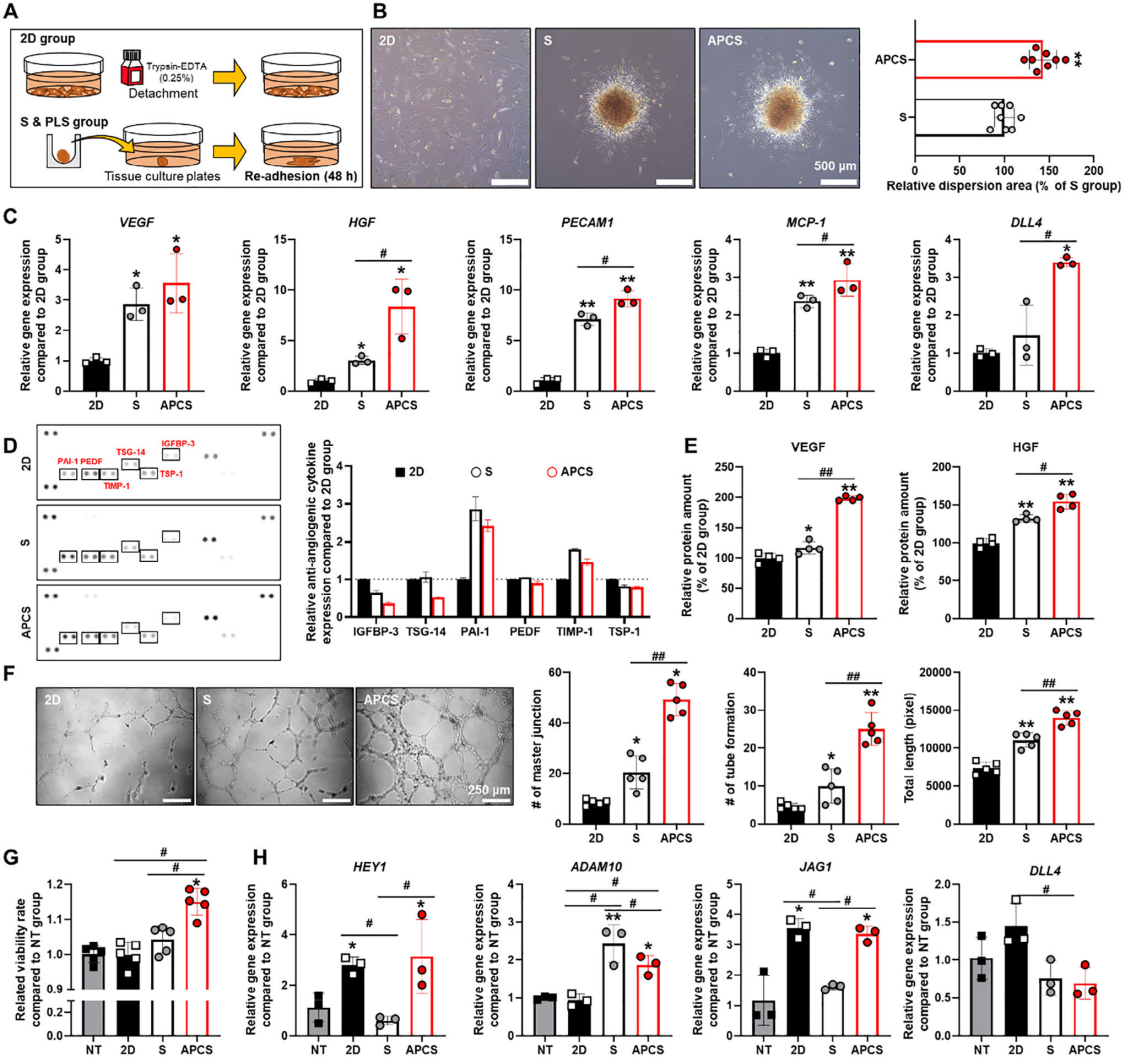
Enhanced vascularization efficacy of reinforced hADSC spheroids. A) Cell adhesion was analyzed 48 h after cell attachment to investigate the dispersion ability of hADSC spheroids. B) Relative dispersion area. The S group was used as the control (*n* = 8, ^*^ *p* < 0.05 vs S group). C) Relative expression of angiogenesis related genes in hADSCs that were reattached for 48 h. The 2D group was used as the control (*n* = 3, ^*^ *p* < 0.05 vs 2D group, ^#^ *p* < 0.05 vs each group). D) Expression of anti‐angiogenesis related proteins in hADSCs analyzed using human angiogenesis array. Representative image of the human angiogenesis array data and comparison of quantitative difference. E) Angiogenesis related factors secreted by hADSCs in the conditioned medium evaluated using ELISA (*n* = 4, ^*^ *p* < 0.05 vs 2D group, ^#^ *p* < 0.05 vs each group). F) HUVEC tube formation assay after incubating HUVECs with conditioned medium retrieved from the hADSCs with quantification data (*n* = 5, ^*^ *p* < 0.05 vs 2D group, ^#^ *p* < 0.05 vs each group). G) Relative cell viability of the HCMECs in hypoxia evaluated using CCK‐8 (*n* = 5, ^*^ *p* < 0.05 vs NT group, ^#^ *p* < 0.05 vs each group). H) Relative expression of HEY1, ADAM10, JAG1, and DLL4 in HCMECs. (*n* = 3, ^*^ *p* < 0.05 vs NT group, ^#^ *p* < 0.05 vs each group).

Next, we performed an attachment experiment to mimic the in vivo ischemic microenvironment after cell transplantation (**Figure** **3**A–C). Cells in the 2D group were collected using trypsin, and cells in the spheroids groups (S and APCS) were collected from intact spheroids (Figure 3A). A comparison of the dispersion area between the spheroid groups (S and APCS) showed that the APCS group spread approximately 150% wider than that of the S group (Figure 3B). Analysis of gene expression in the readhered cells (Figure 3C) showed that *VEGF* expression was higher in the S and APCS groups than that in the 2D group. Additionally, the expression of *HGF*, which improves angiogenesis, and that of *PECAM1* and *MCP‐1*, which improve arterialization, was higher in the APCS group than in the other groups.^[^ ^37^ ^]^ *DLL4* expression was higher in the APCS group than in the other groups. The therapeutic action of stromal cell transplantation is generally mediated by various paracrine factors secreted by the cells after transplantation.^[^ ^16^, ^38^ ^]^ Analysis of the paracrine factors secreted by these cells showed that the levels of anti‐angiogenic factors in the conditioned medium (CM) collected from the APCS groups were lower than those in the CM collected from the S group (Figure 3D). Additionally, VEGF and HGF concentrations in the CM of the APCS group were significantly higher than those of the other groups (Figure 3E). We then performed a tube formation assay using HUVECs to confirm the angiogenic effect of paracrine factors in the CM and found that cells treated with CM from the APCS group exhibited more junctions and tube formation than those from the other groups (Figure 3F).^[^ ^39^ ^]^ Additionally, HCMECs, which play a major role in arterialization,^[^ ^40^ ^]^ were treated with CM in a hypoxic chamber (2% O_2_) to mimic the in vivo ischemic disease conditions HCMECs viability improved 1.1‐fold on treatment with CM from the APCS group compared with the S group (Figure 3G). The no treatment (NT) group was maintained in basal medium without CM. We postulated that the paracrine factors secreted by the APCS group cells had affected the expression of Notch signaling‐related genes in the HCMECs (Figure 3H). Consequently, the activation of Notch signaling in the HCMECs treated with CM from the APCS group increased the expression of *HEY1* and ADAM10 in the APCS group compared with the other groups. Additionally, JAG1 expression increased, whereas that of DLL4 decreased in the HCMECs treated with CM from the APCS group.^[^ ^41^ ^]^ In summary, the APCS group showed increased angiogenesis and arterialization related gene and protein expression than the other groups. Furthermore, paracrine factors secreted by the APCS group cells stimulate endothelial cells to induce angiogenesis and arterialization.

### 2.4 Improved Therapeutic Efficiency in Reinforced Spheroids

**Figure 4:**
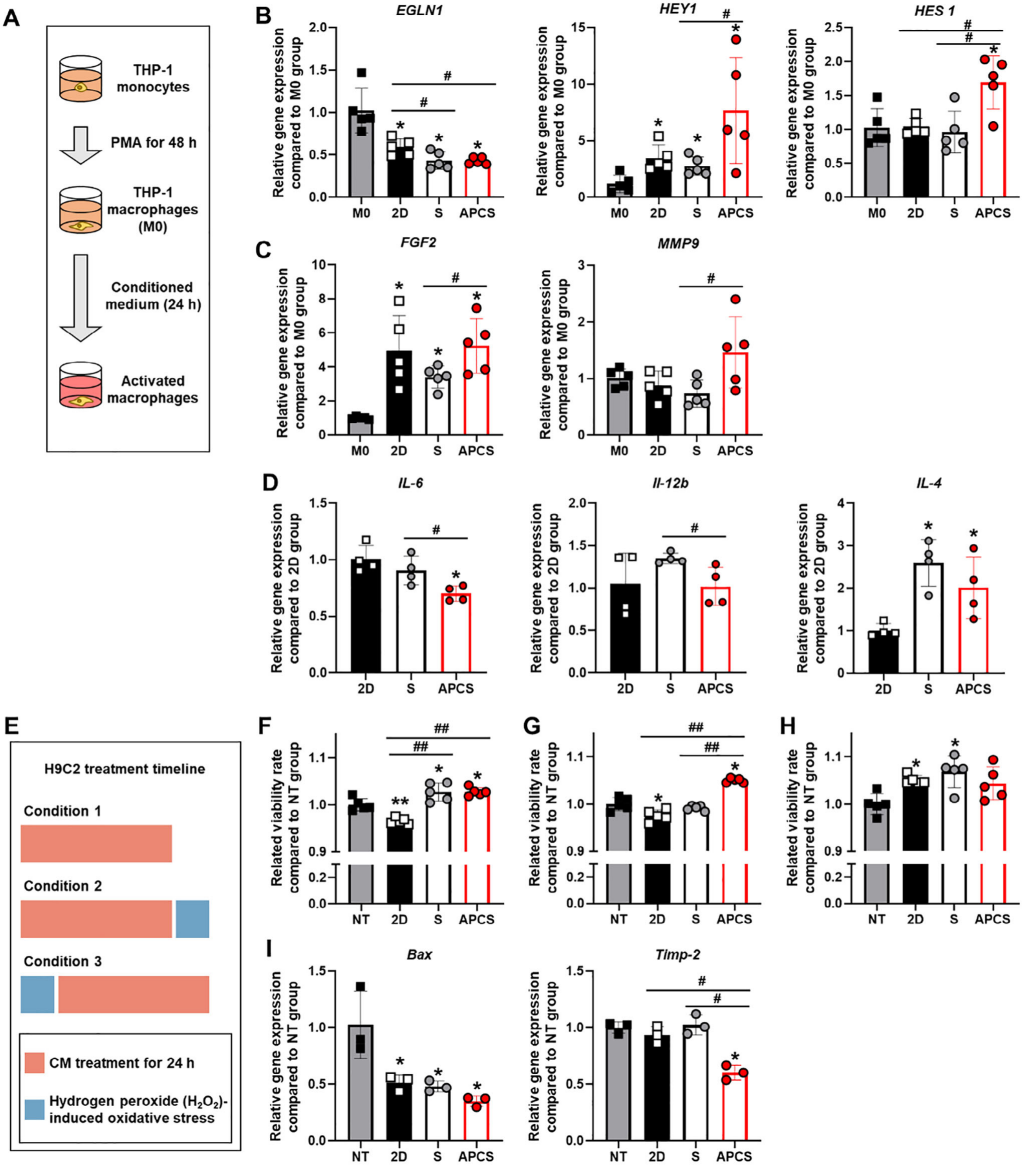
Enhanced paracrine efficacy of reinforced hADSC spheroid. A) Schematic showing the differentiation of THP‐1 cells into macrophages with conditioned medium retrieved from the hADSCs. (B) Relative expression of *EGLN1*, *HEY1*, and *HES1* in THP‐1 cells. The M0 group was used as the control. (*n* = 5, ^*^ *p* < 0.05 vs 2D group, ^#^ *p* < 0.05 vs each group). C) Relative expression of FGF2 and MMP‐9 in THP‐1 cells. The M0 group was used as the control. (*n* = 5, ^*^ *p* < 0.05 vs 2D group, ^#^ *p* < 0.05 vs each group). D) Relative expression of IL‐6, IL‐12b, and IL‐4 in THP‐1 cells. The 2D group was used as the control. (*n* = 4, ^*^ *p* < 0.05 vs 2D group, ^#^ *p* < 0.05 vs each group). E) Conditioned medium retrieved from the hADSCs protects H9C2 cells against H_2_O_2_ induced apoptosis. The relative cellular viability of the H9C2 cells was evaluated using CCK‐8: F) condition 1, G) condition 2, and H) condition 3 (*n* = 5, ^*^ *p* < 0.05 vs NT group, ^#^ *p* < 0.05 vs each group). I) Relative expression of Bax and Timp‐2 in H9C2 cells (condition 3). The NT group was used as the control (*n* = 3, ^*^ *p* < 0.05 vs NT group, ^#^ *p* < 0.05 vs each group).

We next investigated the effect of CM on macrophages, another important cell type that is rapidly recruited to the diseased and has an important role in remodeling.^[^ ^42^ ^]^ As shown in **Figure** **4**A, CM that was collected from each group was applied to the THP‐1 macrophages (M0). The expression of *EGLN1* (also known as PHD2) reduced in all experimental groups compared with that of the control group (M0) (Figure 4B).^[^ ^43^ ^]^ In contrast, we also examined the expression of the Notch signaling‐related factors (*HEY1* and *HES1*) and found both increased in the APCS group compared with the other groups. Notch signaling‐activated macrophages are known to support arteriogenesis.^[^ ^44^ ^]^ The expression of remodeling‐related factors, such as *FGF2* and *MMP‐9*, both of which alter angiogenesis and arterialization, were increased in the APCS group compared with the S group (Figure 4C).^[^ ^45^ ^]^ An examination of macrophage polarization showed that the expression of the inflammatory markers *IL‐6* and *IL‐12b* was lower in the APCS group than that of the other groups (Figure 4D).^[^ ^45^, ^46^ ^]^ The expression of the anti‐inflammatory marker *IL‐4* was higher in the S and APCS groups than in the 2D group. Taken together, APCS CM‐treated macrophages may polarize toward promoting vascularization and reducing inflammation.

Next, we investigated the effects of paracrine factors from the APCS group on cardiomyocytes. H9C2 cells viability was evaluated under various conditions (Figure 4E–H). Under normoxic condition, CM‐treated H9C2 cell viability was increased in the S and APCS groups compared with that observed in the no treatment (NT) and 2D groups (Figure 4F). Similarly, under application of oxidative stress immediately after CM treatment, the H9C2 cells showed enhanced cellular viability in the APCS groups than in the other groups (Figure 4G), indicating activation of protective pathways. However, application of oxidative stress to the H9C2 cells before CM treatment (condition 3), enhanced cell viability in all treatment groups compared with that of the NT group; no difference was observed between the experimental groups (Figure 4H). To further investigate the effects of CM under condition 3, we analyzed gene expression. Gene expression analysis showed that the expression of the proapoptotic factor *Bax* was lower in the APCS group than in the other groups (Figure 4I).^[^ ^47^ ^]^ Additionally, *Timp‐2*, which inhibits angiogenesis when upregulated under hypoxic conditions, was significantly lower in the APCS group than in the other groups (Figure 4I).^[^ ^2^ ^]^ Taken together, the various paracrine factors secreted by the APCS group cells potentially support the myocardial infarction region by activating macrophages and preventing cardiomyocyte apoptosis.

### 2.5 OPBM‐hADSC Spheroids Improve Cardiac Contractility and Remodeling in the Infarcted Hearts

**Figure 5:**
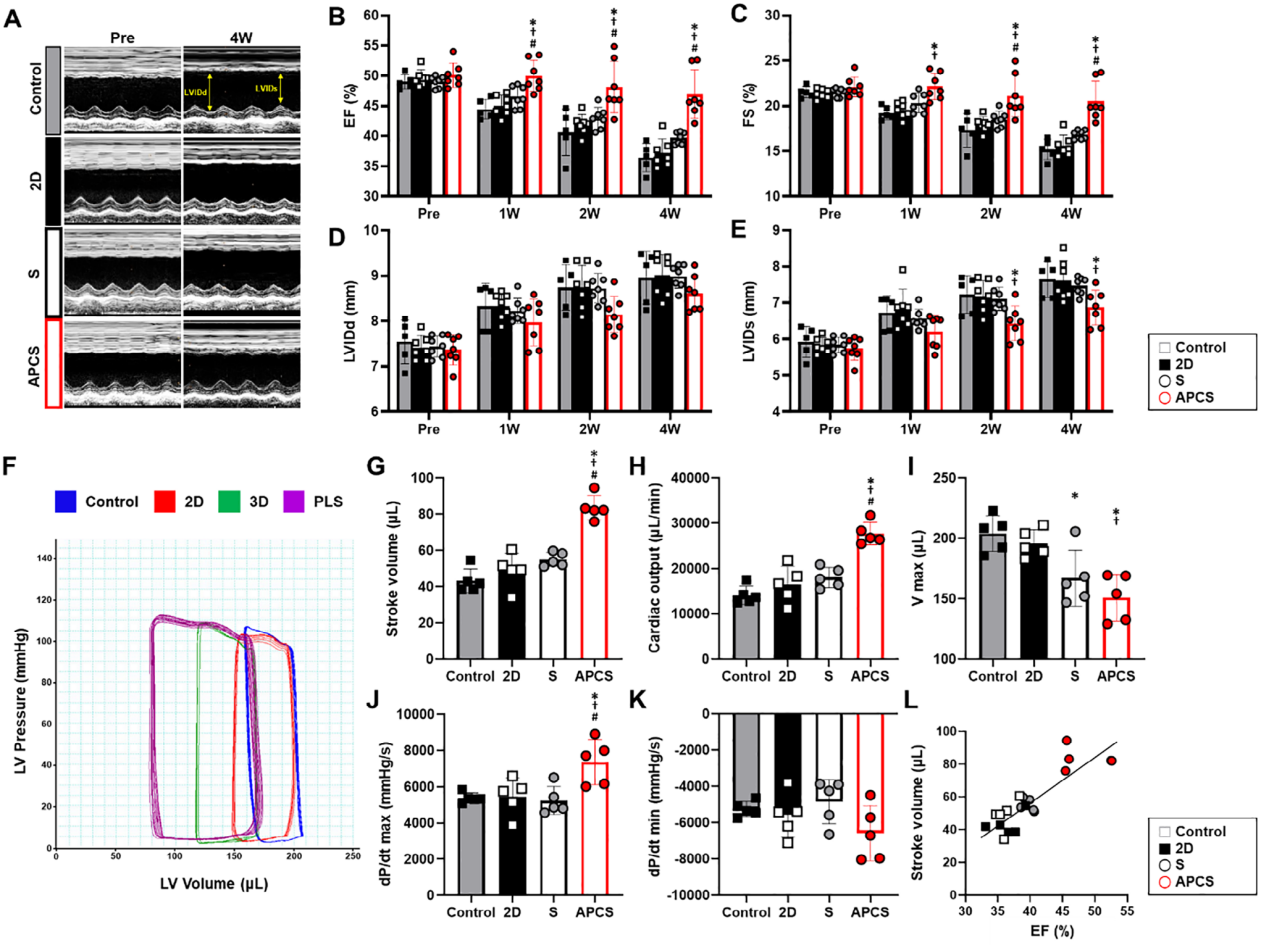
Reinforced hADSC spheroids enhanced cardiac function. A) Representative echocardiography M‐mode images at pre (1‐week post‐MI but prior to intervention) and four weeks post transplantation of reinforced hADSC spheroids. B) Left ventricular ejection fraction (EF). C) Left fractional shortening (FS). D) Left ventricular internal diastolic dimension (LVIDd). E) Left ventricular internal systolic dimension (LVIDs). (*n* = 5–7, ^*^ *p* < 0.05 vs control, ^†^ *p* < 0.05 vs 2D, ^#^ *p* < 0.05 vs S group). F) Representative images of hemodynamic pressure and volume (PV) curve on steady state at four weeks post interventions. G) Stroke volume (SV). H) Cardiac output (CO). I) Volume max (V max) at end‐diastole. J) Maximum rate of pressure changes during systole (dP/dt_max_). K) Minimum rate of pressure change during diastole (dP/dt_min_). L) The slope of correlation between EF and SV. (*n* = 5, ^*^ *p* < 0.05 vs control, ^†^ *p* < 0.05 vs 2D, ^#^ *p* < 0.05 vs S group).

We next investigated the therapeutic effect of the reinforced hADSC spheroids (APCS group) on cardiac function after the transplantation into the infarcted hearts. Ischemia‐reperfusion injury was induced in a rat model by occluding the LAD artery for 1 h, followed by reperfusion. One week after the ischemia‐reperfusion injury, baseline cardiac function was assessed using echocardiography, revealing similar ejection fractions (EF) and fractional shortening (FS) across all groups. Various cell types were then injected into the border zone of infarcted hearts, depending on the presence of photomodulation and 3D spheroid culture. In serial echocardiography, the APCS group showed greater improvements in EF and FS compared to the control and other treatment groups (**Figure** **5**A–C). Additionally, the left ventricular internal diameter at end‐diastole (LVIDd) and left ventricular internal diameter at end‐systole (LVIDs) were also lower in the APCS group than those in the other groups. These findings suggested the light‐preconditioned hADSC spheroids provided more favorable therapeutic effect on cardiac repair by alleviating adverse remodeling post‐MI (Figure 5D,E).

To determine cardiac contractility with hemodynamic pressure and volume during systole and diastole, we directly inserted pressure–volume (PV) catheter into the LV and measured hemodynamic parameters four weeks after the transplantation (Figure 5F). Stroke volume (SV) and cardiac output (CO) were significantly higher in the APCS group than those in the other groups (Figure 5G,H). Additionally, maximum diastolic volume (V_max_) was significantly lower, and dP/dt_max_ and dP/dt_min_, were significantly higher in the APCS groups compared to the other groups, although the maximum pressure (P_max_) was similar in all groups (Figure 5I–K). Two representative parameters of LV systolic function such as EF in echocardiography and SV in PV curve were well correlated according the treatment groups. From these findings, we confirmed that the transplantation of light‐preconditioned hADSC spheroids could improve cardiac function not only by enhancing intrinsic contractility of LV, but also reducing adverse remodeling post‐MI. The EF (%), which was measured using echocardiography, and SV, which was measured using the PV loops, showed a positive correlation (Figure 5L).

### 2.6 Light‐Preconditioned hADSC Spheroids Prevent Deposit of Denature Collagen and Increase Cardiomyocytes Survival

**Figure 6:**
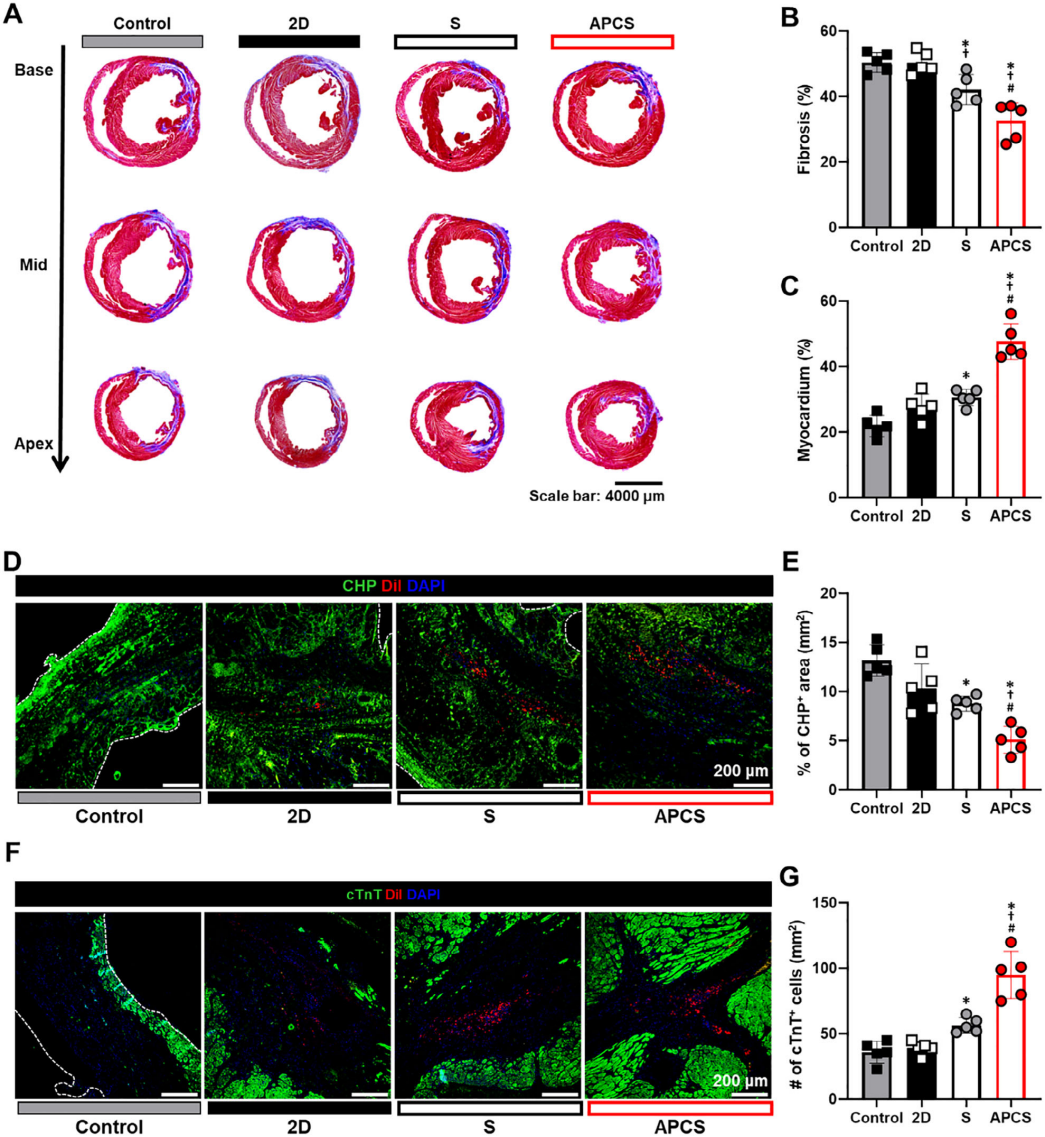
Reinforced hADSC spheroids improved cardiac repair by reducing cardiac fibrosis and protecting the myocardium. A) Representative images of Masson's trichrome staining using the heart tissues harvested four weeks after the intervention. B) Quantification of fibrosis percentage. C) Quantification of myocardium percentage as evaluated based on Masson's trichrome staining. D) Representative images of denatured collagen stained with CHP (green), DiI‐labeled hADSCs (red), and DAPI (blue). E) Quantification of CHP positive area (percentage). F) Representative images of cardiomyocytes stained with cTnT (green), DiI‐labeled hADSCs (red), and DAPI (blue). G) Quantification of the number of cTnT positive cardiomyocytes. (*n* = 5, ^*^ *p* < 0.05 vs control, ^†^ *p* < 0.05 vs 2D, ^#^ *p* < 0.05 vs S group).

We considered that these hemodynamic benefits of light‐preconditioned hADSC spheroids in vivo might include not only the stimulation of angiogenesis to supply oxygen and nutrients to viable myocardium, but also suppression of fibrosis to prevent adverse cardiac remodeling. First, to determine whether the changes of cellular function in OPBM–hADSCs could reduce cardiac fibrosis, we analyzed percent fibrosis in the heart tissues four weeks after transplantation. Indeed, Masson trichrome staining (MT staining) showed that APCS group exhibited significantly lower fibrosis and higher viable myocardium than those of the other groups (**Figure** **6**A–C). To assess the quantity of denatured collagen and cardiomyocytes survival in infarcted hearts, we employed fluorescence‐conjugated collagen hybridizing peptide (CHP) for targeting unfolded collagen chains as well as cTnT staining for visualization. APCS group significantly exhibited not only higher number of total cTnT^+^ CMs, but also reduced denatured collagen deposit in infarcted area compared with those in the control and other treatment groups (Figure 6D–G). These findings confirmed that light‐preconditioned hADSC spheroid reduced fibrosis through preventing deposit of denature collagen and increasing cardiomyocytes survival.

### 2.7 Light‐Preconditioned hADSC Spheroids Plays a Role in the Process of Arterialization to Improve Blood Flow to the Infarcted Hearts

**Figure 7:**
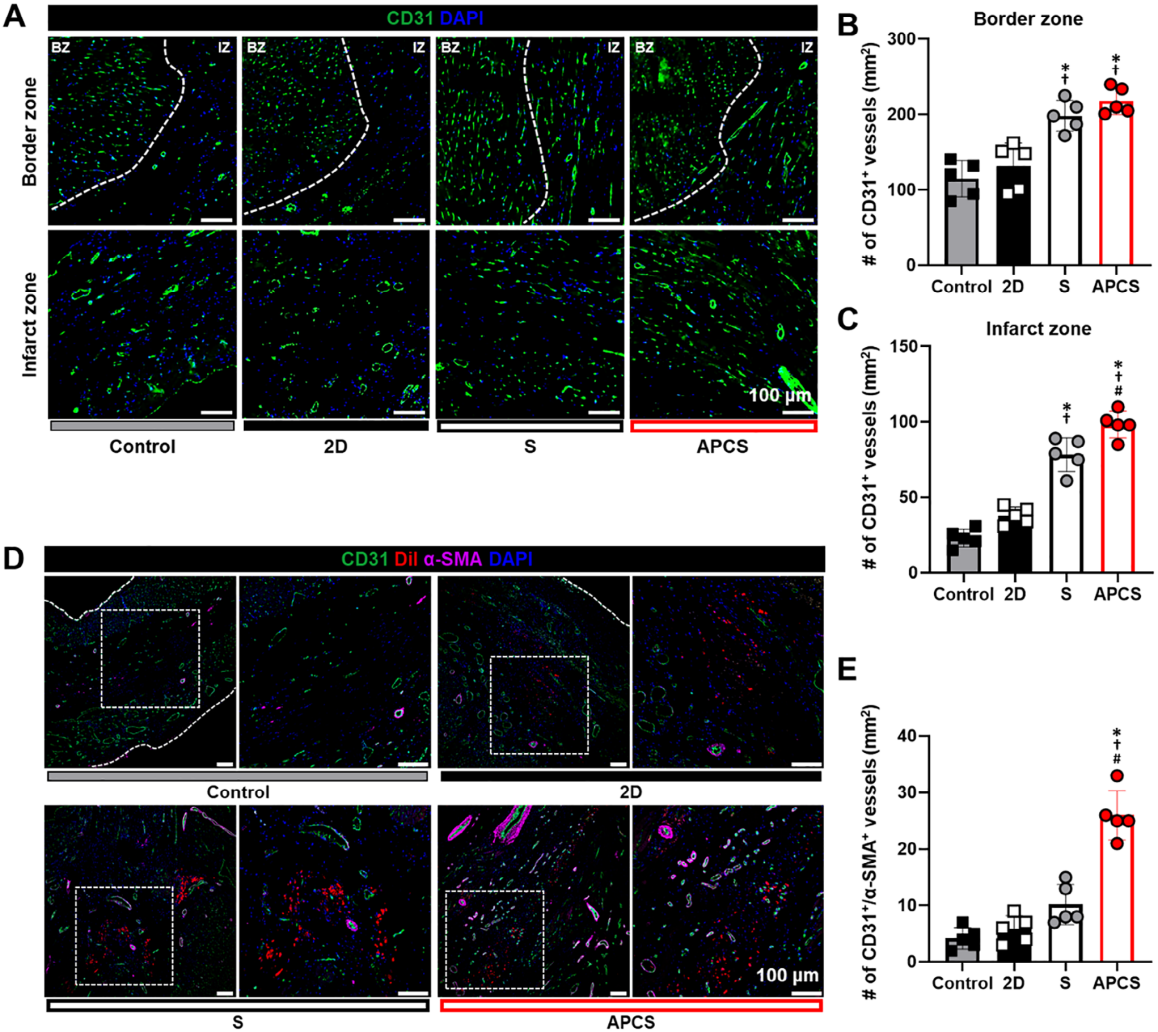
Reinforced hADSC spheroids improved vascular regeneration based on angiogenesis and arterialization. A) Representative images of blood vessels stained with CD31 (green) and DAPI (blue) on the border and infarct zone. B,C) Quantification of the number of vessels in the (B) border and (C) infarct zone. D) Representative images of arteriole‐like vessels stained with CD31 (green), DiI‐labeled hADSCs (red), α‐SMA (purple) and DAPI (blue). E) Quantification of the number of CD31 and α‐SMA positive vessels. (*n* = 5, ^*^ *p* < 0.05 vs control, ^†^ *p* < 0.05 vs 2D, ^#^ *p* < 0.05 vs S group).

Next, we performed CD31 immunohistochemical staining to quantify capillary densities in the border and infarct zone of the hearts. The number of CD31^+^ capillaries in the both border and infarct zone of the hearts was substantially higher in the APCS group than those in the control and the other treatment groups (**Figure** **7**A–C). Notably, the percentage of CD31^+^ α‐SMA^+^ arterioles was also significantly higher in the APCS group than in the other treatment groups, although DiI‐labeled transplanted cells were not incorporated into the host vascular networks but were instead located in the perivascular area (Figure 7D,E). These findings suggested that light‐preconditioned hADSCs were involved mainly in the process of arterialization (defined as process where a vein or capillary bed adopts characteristics more similar to those of arteries) but not in de novo vasculogenesis (defined as recruiting endothelial progenitor cells to foci of neovascularization where they form new blood vessels in situ).

## 3 Discussion

In this study, we explored the potential of OPBM preconditioning to enhance the angiogenic and arterialized capabilities of hADSC spheroids. The in vitro findings indicate that this approach using APCS improved angiogenesis, arterialization, and tissue remodeling efficacy beyond those observed with traditional 2D cultures and standard spheroids (S group). Moreover, this novel strategy to amplify therapeutic effects for cardiac repair showed a notable increase in the efficacy of OPBM‐treated hADSCs compared with that observed in the untreated control (Figure 1A). These results underscore the potential of OPBM preconditioning as a pivotal innovation in stromal cell therapy, paving the way for more effective treatment of cardiac diseases.

ADSCs are considered a promising cell source for cell‐based cardiac repair therapy, because they are easy to obtain and expand from abundant adipose tissue source, are capable of differentiating into multiple cell lineages, and exert several paracrine effects on cardiac repair.^[^ ^3^ ^]^ Although preclinical studies have reported cardiac repair via transplantation of hADSCs in terms of angiogenesis, anti‐inflammation, and cardiomyocyte protection, a significant improvement in cardiac function and blood flow recovery is yet to be reported.^[^ ^4^ ^]^ Researchers have used 3D stromal cell culture to address this limitation in clinical trials. Their function is enhanced mostly due to cell–cell and cell–ECM interactions. These interactions foster the natural behavior of stromal cells, which usually exist in clustered niches, by enhancing their paracrine signaling abilities essential for cardiac repair.^[^ ^17^ ^]^ However, amplifying the therapeutic outcomes becomes further challenging because attempting to enhance hypoxia within these constructs may compromise cell survival and nutrient delivery. This dilemma underscores the difficulty of increasing spheroid size to enhance hypoxia as it might limit the therapeutic efficacy of the cells. Additionally, technical complexity and increased costs are associated with their maintenance and scaling.^[^ ^9^ ^]^

Our study proposes a simplified method to maximize the therapeutic efficiency of 3D cells. In our previous study, OPBM induces an increase in intracellular ROS concentration, which stimulates the RTK pathway in hADSCs.^[^ ^10^ ^]^ This process triggered the enhancement of *HIF‐1α* expression in hADSCs and successively increases the expression of *VEGF*, *VEGFR*, and *HEY1* (Figure 1C).^[^ ^18^ ^]^ The induction of cells to agglomerate on a non‐adhesive plate for 48 h activates cell—to–cell interactions, enhances paracrine and autocrine effects, and improves AKT signaling, NOTCH signaling, and arterialized efficacy of the APCS group cells (Figure 1E,G). We postulate that OPBM induced hASDCs to secrete higher quantities of angiogenic growth factors such as VEGF through the mediation of intracellular ROS that was produces during preconditioning and aggregation, resulting in the higher expression of the earlier‐mentioned factors in the APCS group.^[^ ^48^ ^]^ Also, it appears that activation of these signaling pathways can be maintained by culturing preconditioned hADSCs in a 3D form. During cell aggregation, angiogenesis‐related factors trigger increased *NOX‐4* expression, which induces intracellular ROS.^[^ ^49^ ^]^ Thus, angiogenic factors are further enhanced through increased intracellular ROS levels, and the expression of proapoptotic and profibrotic factors in the APCS group is inhibited (Figure 2).^[^ ^50^ ^]^ Reportedly, Notch signaling was better activated in cardiac progenitor cell (CPC) spheroids than in monolayer cell culture, and signaling was enhanced than under normoxic conditions when CPC spheroids were exposed to a hypoxic environment.^[^ ^17^, ^51^ ^]^ We observed that the expression of *VEGF*, *COX‐2*, and *CXCR4* did not increase when the preconditioned hADSCs were cultured in a 2D form for 48 h (Figure S2, Supporting Information). Furthermore, the APCS group had a more compact and denser spheroid form than that of the S group (Figure 1B). Possibly, the improved intracellular ROS level that was maintained during cell aggregation may have improved the structure of the spheroid by increased integrin αV expression (Figure 1D–F).^[^ ^52^ ^]^ The JAG1, DLL1, and DLL4 Notch receptors were expressed on the surface of the hADSCs. Among them, *DLL4* expression was enhanced in the APCS group (Figure 1G).^[^ ^22^ ^]^ *DLL4* stimulates Notch1 signaling in endothelial cells and enables tip cell migration or stalk cell proliferation.^[^ ^41^ ^]^ These processes play critical roles in angiogenesis and arterialization.^[^ ^40^ ^]^ Transplantation of Notch signaling‐modified MSCs into a hindlimb ischemia model improved therapeutic effect through arterialization 28 d after its introduction. This can be attributed to the activation of Notch signaling in endothelial cells by Notch signaling‐modified MSCs.^[^ ^15b^ ^]^

In this study, we observed that the effect of HIF‐1α signaling after hypoxia‐preconditioning (the HPCS group) was similar to that of OPBM‐preconditioning (the APCS group) (Figure 2A–C). A previous study fabricated spheroids using MSCs that underwent short‐term hypoxia for application in bone regeneration and demonstrated that the osteogenic potential and VEGF secretion of spheroids improved through hypoxia‐preconditioning and that the transplanted spheroids exerted enhanced bone regeneration effects than those of conventional spheroids.^[^ ^8^ ^]^ Similar to that of the HPCS group, the treatment efficiency of the APCS may have been initiated and reinforced by hypoxia at the time of preconditioning. We postulate that angiogenesis was enhanced owing to the hypoxic environment of the spheroids (APCS and HPCS group).^[^ ^53^ ^]^ However, the persistence of the APCS groups’ preconditioning effect appears to be different from that of the HPCS group. This indicates that preconditioning with light may provide a more lasting cellular effect than with hypoxia. Particularly, the APCS group showed a greater change in the expression of *SART1* and *COL15* than that in the S and HPCS groups (Figure 2B,C). Reduced SART1 expression increases VEGF secretion and decreases profibrotic responses in hypoxic lesions.^[^ ^27^ ^]^ Additionally, COL15 is essential for modeling the damaged cardiac extracellular matrix and is primarily involved in microvessel fabrication.^[^ ^28^ ^]^ Preconditioning with OPBM also enhanced the expression of proangiogenic factors and decreased the expression of proapoptotic and profibrotic factors in hADSC spheroids (Figure 2D–J). Particularly, *CD55*, *CXCR4*, and CX43 expression increased and *P16*, *TGF‐ β1*, and β‐catenin expression decreased. Enhanced expression of CXCR4 could be attributed to the enhancing effect of activated AKT signaling.^[^ ^54^ ^]^

Next, we performed in vitro experiments to predict the potential cardiovascular treatment efficiency of the transplanted cells (Figures 3, 4). Increased integrin αV expression induced wider dispersion in the APCS group. Additionally, the expression of angiogenesis‐, arterialization‐, and Notch signaling‐related factors improved adherence in the APCS group (Figure 3A–C). Based on these results, we predict higher engraftment rate and treatment efficiency in the transplanted APCS group than that of other groups. Subsequently, we analyzed the paracrine effects induced by subjecting various types of cells constituting the cardiac microenvironment to treatment with CM. The CM collected from the APCS group reduced the expression of anti‐angiogenic factors (Figure 3D) and also significantly increased the expression of VEGF and HGF compared with that of the other groups (Figure 3E). According to a previous study, transplanting MCSs that have been primed to continuously secrete HGF improved cardiac function and vessel formation in an MI model.^[^ ^55^ ^]^ In addition to improving HUVEC angiogenesis (Figure 3F), APCS CM enhanced the viability of the hCMECs exposed to a hypoxic environment (Figure 3G). Moreover, APCS CM induced hCMECs to become stalk‐competent cells by regulating hCMEC Notch signaling (Figure 3H). These stalk cells play a major role in shaping the vessel lumen and trunk.^[^ ^56^ ^]^ Furthermore, the decreased *EGLN1* expression that was observed in the THP‐1 macrophages treated with APCS CM may have promoted macrophage polarization and aided in arterialization (Figure 4B).^[^ ^42^, ^43^, ^45^ ^]^ APCS CM also regulates THP‐1 macrophages through Notch signaling, which may have aided macrophage maturation in the ischemic environment (Figure 4B).^[^ ^44^ ^]^ Additionally, APCS CM upregulated vascularization effect of macrophages and inhibited inflammation (Figure 4C,D). APCS CM treatment improved cell viability by protecting H9C2 from H_2_O_2_ induced oxidative stress (Figure 4F–H). Although the overall improvement in cell viability was less pronounced in condition 3 (Figure 4H), the downregulation of *Bax* and *Timp‐2* suggests that APCS CM exerts effects beyond direct viability enhancement, potentially modulating apoptotic and angiogenic pathways (Figure 4I). These molecular effects may contribute to long‐term survival and functional recovery despite minimal immediate differences in viability. We hypothesize that the effect exerted by the paracrine factors from the APCS group prevents apoptosis and promotes angiogenesis in the ischemic disease‐impacted cardiomyocytes. This comprehensive suite of effects underlines the multifaceted benefits of APCS CM ranging from enhancing angiogenesis and arterialization to modulation of immune response and protection against cellular stress; thus, this study lays a robust foundation for its application in cardiac repair.

Furthermore, this study underscores the potential of light‐preconditioned hADSC spheroids in enhancing cardiac repair and regeneration (Figure 5) while significantly ameliorating cardiac fibrosis (Figure 6) following myocardial infarction (MI). Cardiac fibrosis contributes to cardiac dysfunction by increasing ventricular stiffness, which increases the risk of arrhythmogenesis and mortality.^[^ ^35^ ^]^ In this study, the APCS group showed a reduced accumulation of collagen, which could contribute to the functional repair seen (Figure 6D,E). Transplantation of reinforced hADSC spheroids also improved cardiomyocyte protection (Figure 6F,G) and vascular regeneration (Figure 7). Notably, increased myocardial protection and vascular regeneration occurred in the regions adjacent to the engraftment of DiI‐labeled hADSCs, although direct differentiation of hADSCs into the cardiac component cell lineage in vivo was not observed. Taken together, these findings suggest that the enhancement strategy to amplify the therapeutic effects of hADSC spheroids via light‐preconditioning significantly improved myocardial protection and vascular regeneration while reducing cardiac fibrosis. This approach could accelerate research in the field of stromal cell‐based therapy and regenerative medicine. Furthermore, this study underscores the need to further explore and develop this promising field for ultimately improving patient outcomes in cardiac regeneration therapy. In addition, standard immunosuppressants were administered during the transplantation of human cells into rat hearts. However, the impact of immunosuppressants on the outcomes of this study remains inconclusive. Nevertheless, because the therapeutic effect of stem cell therapy largely depends on the engraftment and survival of the transplanted cells, we believe that immunosuppressive administration may be essential to prevent immune rejection of transplanted cells and allow to the achievement of outstanding therapeutic effects of stem cell‐based therapy.

## 4 Experimental Section

### Cell Culture

hADSCs were obtained from Lonza (Basel, Switzerland) and cultured in Dulbecco's modified Eagle's medium (DMEM, Gibco BRL, Gaithersburg, Maryland, USA) supplemented with 10% (v/v) fetal bovine serum (FBS, Gibco BRL) and 1% (v/v) penicillin/streptomycin (PS, Gibco BRL). Endothelial cells, including HUVECs (purchased from Lonza) and HCMECs (purchased from ScienCell Research Laboratories (San Diego, CA, USA)), were cultured in endothelial cell growth medium‐2 (EGM‐2, Lonza) and endothelial cell medium (ScienCell Research Laboratories), respectively. THP‐1 cells were obtained from ATCC (Manassas, VA, USA) and cultured in RPMI medium 1640 (Gibco BRL) supplemented with 10 FBS and 1% (v/v) PS. Similarly, H9C2 cells were purchased from ATCC and cultured in DMEM (high glucose) supplemented with 10 FBS and 1% (v/v) PS. All cell lines were incubated under standard culture conditions at 37 °C with 5% CO_2_. Media changes were performed every 2 d, and cells were used for experiments prior to passage 6.

### Cell Preconditioning and Aggregation

hADSCs were preconditioned using red OLED (Kaneka, Osaka, Japan) irradiation under the same conditions as previously described, ensuring angiogenic activation. The cells were exposed to light for 24 h at energy densities of ≈300 J cm^−2^, following the protocol established in our prior study.^[^ ^10^ ^]^ After preconditioning, hADSCs were collected using trypsin (Gibco BRL) and resuspended in cell culture medium containing 10 FBS and 1% (v/v) PS. The resuspended hADSCs were seeded onto u‐type 96‐well cell culture plates (SPL life sciences, Gyeonggi‐do, Korea) coated with 2% (v/w) poly(2‐hydroxyethyl methacrylate) (Sigma‐Aldrich, St. Louis, Missouri, USA) and centrifuged for 5 min at 400 × g. Aggregation was induced by culturing 3 × 10^3^ cells 100 µL^−1^ for an additional 48 h.^[^ ^57^ ^]^

### Quantitative Reverse Transcription Polymerase Chain Reaction (qRT‐PCR)

qRT‐PCR was conducted to analyze relative gene expression levels accross experimental groups. Total RNA was extracted from the samples using 1 mL TRIzol reagent (Life Technologies, Inc., Carlsbad, CA, USA) and 200 µL chloroform. Lysates were centrifuged at 12 000 rpm for 10 min at 4 °C, and RNA pellet was washed with 75% (v/v) ethanol in water and dried. The samples were dissolved in RNase‐free water. qRT‐PCR reactions were performed using the SsoAdvanced Universal SYBR Green Supermix kit (Bio‐Rad, Hercules, CA, USA) and the CFX Connect real‐time PCR detection system (Bio‐Rad). The primer sequences used for qRT‐PCR were listed in Table S1 (Supporting Information).

### Intracellular Reactive Oxygen Species (ROS) Detection

Intracellular ROS levels were quantified using the fluorescent ROS indicator DCF (D339 Invitrogen, Carlsbad, CA, USA) and analyzed via fluorescence‐activated cell sorting (MACSQuant VYB; Miltenyi Biotec, Bergisch Gladbach, Germany). hADSC spheroids were dissociated using trypsin and incubated with 2.5 µm DCF in PBS at 37 °C for 10 min to stain intracellular ROS. The stained cells were washed with FACS buffer (BioLegend, San Diego, CA, USA), resuspended in FACS buffer, and analyzed using flow cytometry (MACSQuant VYB, Miltenyi Biotec).

### Western Blotting Analysis

The aggregated hADSC spheroids were cultured for 48 h, collected, and lysed in RIPA buffer (Rockland Immunochemicals Inc., Limerick, PA, USA). After centrifugation at 10 000 × g for 10 min, the supernatant was used as the protein extract for western blotting analysis. Protein concentrations were determined using a BCA assay (Pierce Biotechnology, Rockford, IL, USA). Equal quantities of proteins from each sample were mixed with sample buffer and subjected to sodium dodecyl sulfate polyacrylamide gel electrophoresis (SDS‐PAGE) using a 10% (v/v) resolving gel. The separated proteins were transferred onto immune‐blot PVDF membranes (Bio‐Rad). The membranes were blocked with 5% (w/v) skim milk in Tris‐buffered saline (TBS; 50 mm Tris–HCl (pH 7.5), 150 mm NaCl, and 2.5 mm KCl) and incubated for 1 h at 25 °C. Then, the membranes were probed overnight at 4 °C with antibodies against glyceraldehyde 3‐phosphate dehydrogenase (GAPDH, 1:2000, Abcam, Cambridge, UK), AKT, P‐AKT, INTEGRIN αV, HIF‐1α, NOX‐4, P21, and CX‐43 (Abcam). Next, the membranes were incubated with horseradish peroxidase‐conjugated secondary antibody (R&D Systems) for 1 h at 25 °C, followed by the addition of ECL reagent (TransLab, Daejeon, Korea). The blots were developed in a dark room, and luminescence was recorded using an X‐ray blue film (Agfa HealthCare NV, Mortsel, Belgium).

### Immunocytochemistry (In Vitro)

Spheroid morphology during aggregation was analyzed under a microscope (CKX53; Olympus, Tokyo, Japan). After 48 h of aggregation, the spheroids were fixed with 4% paraformaldehyde (Biosesang, Sungnam, Korea) overnight at 4 °C. The fixed spheroids were embedded in optimal cutting temperature (OCT) compound (Scigen Scientific, Gardena, CA, USA). Frozen samples were sectioned into 10 µm thick slices at −20 °C, and the sections containing the spheroids were stained. Immunofluorescence staining was conducted to detect integrin αV, F‐actin, β‐catenin, and osteopontin. The samples were treated with anti‐integrin αV, TRITC‐phalloidin containing a mounting medium (Vectashield H‐1600, Vector, Burlingame, CA, USA), anti‐β‐catenin, and anti‐osteopontin antibodies, followed by incubation with a fluorescein isothiocyanate (FITC)‐conjugated secondary antibody (1:50, Jackson ImmunoResearch Laboratories, West Grove, PA, USA, 11). The sections were counterstained with DAPI and examined under a fluorescence microscope (DFC 3000 G; Leica, Wetzlar, Germany).

### Cell Viability Assay

The cell viability assay was performed using fluorescein diacetate (FDA, Sigma‐Aldrich) and ethidium bromide (EB, Sigma‐Aldrich). The cytoplasm of viable cells and the nuclei of nonviable cells were stained with FDA (green) and EB (red), respectively. The staining solution was freshly prepared by combining 10 mL of FDA stock solution (1.5 mg mL^−1^ of FDA in dimethyl sulfoxide), 5 mL of EB stock solution (1 mg mL^−1^ of EB in PBS), and 3 mL of PBS. hADSC spheroids were washed once with PBS and incubated with the staining solution for 3 min at 37 °C. After staining, the samples were washed twice with PBS and examined under a fluorescence microscope (DFC 3000 G). The apoptosis assay was performed using the FITC Annexin V Apoptosis Detection Kit with 7‐AAD (BD Biosciences, San Diego, CA, USA) according to the manufacturer's instructions. The hADSC spheroids were dissociated using trypsin‐EDTA (Gibco BRL). The cells were incubated in the dark with FITC Annexin V and 7‐AAD for 15 min at room temperature. After staining, the cells were added to Annexin V binding buffer and analyzed using a flow cytometer (MACSQuant VYB, Miltenyi Biotec, Bergisch Gladbach, Germany). Annexin V and 7‐AAD bound to phosphatidylserine and the nuclei of nonviable cells, respectively. Cells stained as annexin V^−^/7AAD^−^ (live), annexin V^+^/7AAD^−^ (early apoptotic), and annexin V^+^/7AAD^+^ (late apoptotic or earl necrotic) were classified accordingly.

### Re‐Adhesion Test

hADSCs in the 2D group were detached using trypsin and immediately reseeded onto 6‐well plates. In contrast, hADSC spheroids were directly reseeded onto the 6‐well plates without dissociation. Both cells and spheroids were attached to conventional tissue culture plates and incubated for 48 h. Unattached cells were removed by washing with PBS. After incubation, the cells were observed under a microscope (DFC 3000 G).

### Paracrine Signaling Factor Secretion

The Proteome Profiler Human Angiogenesis Array Kit (R&D Systems, Minneapolis, MN, USA) was utilized to analyze anti‐angiogenic protein expression in the conditioned medium (CM) following the manufacturer's protocol. The pixel density of each spot was quantified using ImageJ software (National Institutes of Health) and the average signal was calculated from duplicate spots. The amounts of VEGF and HGF in the CM were measured using human VEGF DuoSet ELISA (R&D Systems) and human HGF DuoSet ELISA (R&D Systems) in accordance with the manufacturer's instructions. Optical density was recorded at 450 nm (correction at 540 nm) using a microplate reader (Infinite F50, Tecan, Mannedorf, Switzerland).

### Tube Formation Assay

Endothelial cell tube formation was evaluated using an angiogenesis assay kit (Abcam), following the manufacturer's protocol. HUVECs (2 × 10^4^ cells well^−1^) were seeded onto an extracellular matrix gel in 100 µL of medium and incubated for 4 h at 37 °C. After incubation, the HUVECs were stained with the staining dye for 30 min at 37 °C and observed under a fluorescence microscope (IX71, Olympus). Tube formation was quantified using ImageJ (NIH, Bethesda, MD, USA) with the Angiogenesis Analyzer plugin.

### Macrophage Polarization

THP‐1 cells were purchased from American Type Culture Collection (Manassas, VA, USA) and cultured in cell culture flasks (Corning) using RPMI‐1640 medium (Gibco BRL), supplemented with 10 FBS (Gibco BRL) and 1% (v/v) PS (Gibco BRL), in a 5% CO_2_ cell incubator at 37 °C. The culture medium was replaced every 2 d. THP‐1 cells at passages 2–5 were used for the experiments. To induce differentiation, THP‐1 cells were treated with 100 nm mL^−1^ phorbol 12‐myristate 13‐acetate (PMA, Sigma‐Aldrich) for 2 d. The cells were washed with PBS and polarized toward the M1 or M2 phenotype by incubation for 2 d in a sample medium.

### hADSC Staining

For in vivo cell‐fate tracking, hADSCs were labeled with CellTracker CM‐1,1′‐dioctadecyl‐3,3,3′,3′‐tetramethylindocarbocyanine perchlorate (DiI) (Invitrogen). Labeling was performed by suspending the cells (1 × 10^6^ cells mL^−1^) in DPBS and adding 1 µm mL^−1^ DiI. After incubation for 15 min at 37 °C, the cells were centrifuged at 1500 rpm for 5 min and washed twice with DPBS. To prepare the cells for transplantation, the DiI‐labeled hADSCs (1 × 10^6^) were suspended in 100 µL of saline.

### Ischemia‐Reperfusion Modeling Model and Spheroid Transplantation

All animal experiments were approved by the Institutional Animal Care and Use Committee (IACUC) of The Catholic University of Korea (approval number: CUMC‐2020‐0051‐01). All procedures conformed to the guidelines of the Directive 2010/63/EU of the European Parliament on the protection of animals used for scientific purposes or the NIH guidelines. Fischer 344 rats (160–180 g, male, Koatec, Korea) were anesthetized with 2% inhaled isoflurane and intubated via the trachea using an 18‐gauge intravenous catheter. The rats were mechanically ventilated using a rodent respirator (55–7058, Harvard Apparatus, Canada) and placed on a 37 °C heating pad to maintain body temperature during the procedure. Left thoracotomy was performed after shaving the chest and sterilizing with 70% alcohol. Ischemia‐reperfusion injury was induced by occluding the LAD artery with a 7‐0 Prolene suture for 1 h. Baseline left ventricular function was assessed by examining the ejection fraction (EF) on post‐operative day (POD) 7. The rats were re‐anesthetized the following day using isoflurane inhalation, intubated, and mechanically ventilated. The animal chest was reopened and spheroids were injected at two sites in the border zone of the infarcted heart. The following four experimental groups were established: 1) MI control; 2) 2D (1 × 10^6^ cells); 3) S (1 × 10^6^ cells); and 4) APCS (1 × 10^6^ cells). The chest was closed aseptically, and antibiotics and 0.9% normal saline solution were administered. All rats received daily immunosuppressants as described previously: azathioprine, 2 mg kg^−1^; cyclosporine A, 5 mg kg^−1^, and methylprednisolone, 5 mg kg^−1^.

### Echocardiography

The animals were lightly anesthetized with isoflurane and maintained at 37 °C using a heating pad. Functional improvement in the injured cardiac tissues was assessed via echocardiography. The rats were anesthetized with isoflurane inhalation, and physiological data were recorded using a transthoracic echocardiography system equipped with a 15 MHz L15‐7io linear transducer (Affniti 50G, Philips, Netherlands). Serial echocardiography was conducted before and 1, 2, and 4 weeks after treatment. The echocardiography operator was blinded to group allocation during the experiment. The ejection fraction (EF) and fractional shortening (FS) indices of LV systolic function were calculated as following equations:

(1) $$-\mathrm{Vd}=\mathrm{LVID}d^{3},\mathrm{Vs}=\mathrm{LVID}d^{3}$$

(2) $$-\mathrm{EF}\left(\%\right)=\left(\mathrm{Vd}-\mathrm{Vs}\right)/\mathrm{Vd}$$

(3) $$-\mathrm{FS}\left(\%\right)=100\times (\mathrm{LVIDd}-\mathrm{LVIDs})/\mathrm{LVIDd}$$

where LVIDd and LVIDs represented the left ventricular internal diameter at end‐diastole and end‐systole, respectively.

### Hemodynamic Measurements

Hemodynamic measurements were conducted at the end of 8 weeks before euthanasia. After performing, a thoracotomy without bleeding, the LV apex of the heart was punctured with a 26‐gauge needle, through which a 2F conductance catheter (SPR‐838, Millar, USA) was inserted into the LV. LV pressure–volume (PV) parameters were continually recorded using a PV conductance system (MPVS Ultra, EMKA Technologies, France) coupled with a digital converter (PowerLab 16/35, ADInstruments, New Zealand). Load‐independent cardiac function measurements, including the slopes of the end‐systolic pressure‐volume relationship (ESPVR) and end‐diastolic pressure‐volume relationship (EDPVR), were obtained by altering preload conditions through transient inferior vena cava (IVC) occlusion with a needle holder. To calculate parallel conductance after hemodynamic measurements, a 50 µL aliquot of hypertonic saline (20% NaCl) was injected into the left jugular vein. Blood was collected from the left ventricle using a heparinized syringe and placed in cuvettes to convert the conductance signal into volume using a catheter. The absolute blood volume of the rats was determined by calibrating the parallel conductance and the cuvette conductance.

### Determination of Fibrosis

Circumferential fibrosis and viable myocardium were assessed using Masson's trichrome staining (Sigma, St. Louis, MO, USA). Briefly, three paraffin slides were preincubated at 37 °C in a dry oven before deparaffinization and rehydration. The paraffin sections were then refixed for 1 h at 56 °C in Bouin's solution. These sections were initially stained with Weigert's iron hematoxylin solution for 15 min at room temperature, followed by staining with Biebrich scarlet‐acid fuchsin solution for 20 min. Finally, the sections were counterstained with aniline blue for 15 min, and incubated in 1% acetic acid for 1 min. Extensive washing was performed between the steps. The heart sections were imaged using a slide scanner (Pannoramic MIDI), and the fibrosis ratio was calculated as the area of fibrosis to the area of the LV circumference ([infarct area/LV wall area] × 100). Additionally, the ratio of the myocardium was quantified as the area of the myocardium within the infarct area after dilating the LV wall, and observing the fibrotic area: ([myocardial area/infarct area] × 100). Both measurements were performed using ImageJ software (National Institutes of Health).

### Immunohistochemistry (In Vivo)

After the animals were euthanized at 4 weeks, the hearts were fixed in 4% paraformaldehyde and embedded in paraffin. The hearts were sectioned into 5 µm sections starting from the top of the apex. The sections were deparaffinized and rehydrated. Antigen retrieval was performed in a humid chamber using a target retrieval solution (S3022, DAKO, Denmark). The sections were incubated overnight at 4 °C with a diluted primary antibody (Dako). The primary antibodies used were anti‐vimentin (Abcam), anti‐CD31 (Novus, USA), anti‐SMA (Abcam), anti‐cTnT (Abcam), and collagen‐hybridized peptide (CHP; 5‐FAM conjugate, 3Helix, USA). After washing three times with 1% Tween 20 in PBS, the sections were incubated in the dark with the secondary antibody for 90 min at room temperature. The following secondary antibodies were used: anti‐rabbit 594 (Invitrogen), anti‐mouse 488 (Invitrogen), and anti‐rabbit 647 (Invitrogen). After washing three times with PBS, the sections were stained with DAPI solution (Vector) for nuclear staining and mounted on slides. The number of capillaries was counted in five random microscopic fields using a fluorescence microscope (ECLIPSE Ts2, Nikon, Japan) and expressed as the number of capillaries per square millimeter of tissue area.

### Statistical Analysis

All statistical analyses were performed using GraphPad Prism 7 software. Triplicate data from all experiments were analyzed using one‐way analysis of variance (ANOVA) with the Bonferroni test. Comparisons between two independent samples were made using the two‐tailed Student's *t*‐test. Statistical significance was defined as *p* < 0.05. All results were presented as the mean ± standard deviation for the quantitative analyses.

## Conflict of Interest

The authors declare no conflict of interest.

## Supporting information



Supporting Information

## Data Availability

The data that support the findings of this study are available from the corresponding author upon reasonable request.
