# Supplementary material for: Reinforcing Stromal Cell Spheroid Through Red‐Light Preconditioning for Advanced Vascularization
Source: Adv Sci (Weinh). 2025 Apr 25;12(29):2500788. doi: 10.1002/advs.202500788 (PMC12362804; doi:10.1002/advs.202500788)
Supplement: Supplementary file 1 — Supporting Information [file ADVS-12-2500788-s001.docx]

Supporting Information

Reinforcing stromal cell spheroid through red-light preconditioning for advanced vascularization

*Yu-Jin Kim^1,2,†^, Hyeok Kim^2,†^, Dong-Hyun Lee^1^, Yeong Hwan Kim^1^, Jae-Hyun Park^3^, Woo-Sup Sim^3^, Jin-Ju Kim^3^, Kiwon Ban^4^, Soong Ho Um^1^, Hyun Ji Park^7^, Michael E. Davis^8,9^ Hun-Jun Park^3,5,6*^, Suk Ho Bhang^1,*^*

*TUNEL staining for apoptotic cell detection* After 48 h of aggregation, the spheroids were fixed with 4% paraformaldehyde (Biosesang, Sungnam, Korea) overnight at 4 °C. The fixed spheroids were embedded in optimal cutting temperature (OCT) compound (Scigen Scientific, Gardena, CA, USA). Frozen samples were sectioned into 10-μm-thick slices at -20 °C, and the sections containing the spheroids were stained. TUNEL staining was performed according to the manufacturer's protocol using an ApopTag^®^ fluorescein in situ apoptosis detection kit (Millipore, Billerica, MA, USA) to assess apoptotic activity. The cells were counterstained with 4,6-diamidino-2-phenylindole (DAPI; Vector Laboratories, Burlingame, CA, USA) and examined under a fluorescence microscope (DFC 3000 G, Leica, Wetzlar, Germany).

**Table S1. qRT-PCR primer sequences (verified using BLAST)**

| Gene | Primer | Sequence (5′-3′) |
| --- | --- | --- |
| *Human GAPDH* | Forward | GTC GGA GTC AAC GGA TTT GG |
|  | Reverse | GGG TGG AAT CAA TTG GAA CAT |
| *Human HIF-1α* | Forward | CAG TTA CGT TCC TTC GAT CAG TTG |
|  | Reverse | TTT GAG GAC TTG CGC TTT CA |
| *Human VEGF* | Forward | GAG GGC AGA ATC ATC ACG AAG T |
|  | Reverse | CAC CAG GGT CTC GAT TGG AT |
| *Human VEGFR* | Forward | CAG CGA TGG CCT CTT CTG TAA |
|  | Reverse | TCC AGT GTC ATT TCC GAT CAC T |
| *Human HEY1* | Forward | ACC TGA AAA TGC TGC ACA CG |
|  | Reverse | GCT GGG AGG CGT AGT TGT TA |
| *Human GSK3B* | Forward | CCG ACT AAC ACC ACT GGA AGC T |
|  | Reverse | AGG ATG GTA GCC AGA GGT GGA T |
| *Human JAG1* | Forward | AAT GGC TAC CGG TGT GTC TG |
|  | Reverse | CCC ATG GTG ATG CAA GGT CT |
| *Human DLL4* | Forward | CTG CGA GAA GAA AGT GGA CAG G |
|  | Reverse | ACA GTC GCT GAC GTG GAG TTC A |
| *Human PCNA* | Forward | CCT GCT GGG ATA TTA GCT CCA |
|  | Reverse | CAG CGG TAG GTG TCG AAG C |
| *Human BICD1* | Forward | TCC ATC CAC CGG AAG GTT G |
|  | Reverse | GGC TCT GTT TCA GCT CGT TC |
| *Human COL15A1* | Forward | CAG TGC TGG TGT CTG CTG AT |
|  | Reverse | GAC AAA GGA TAC GGA CGA GG |
| *Human C-MYC* | Forward | AAT GAA AAG GCC CCC AAG GTA GTT ATC C |
|  | Reverse | GTC GTT TCC GCA ACA AGT CCT CTT C |
| *Human SART1* | Forward | TGG CCT CCG AAT ACC TCA C |
|  | Reverse | CCG CAG TCT GGA ACC AAA G |
| *Human DISC1* | Forward | CAG CAC CCT GAG GAA GAA AG |
|  | Reverse | TAG CCG TCC AGA AAT GGT TT |
| *Human COX-2* | Forward | CCA GCA CTT CAC GCA TCA GT |
|  | Reverse | ACG CTG TCT AGC CAG AGT TTC AC |
| *Human IGF-1* | Forward | CAC AGA CGG GCA TCG TGG AT |
|  | Reverse | ACT TGG CAG GCT TGA GGG GT |
| *Human ANGPT-1* | Forward | GGA AAT CCC TCC GGT GAA TA |
|  | Reverse | GAA TAG GCT CGG TTC CCT TC |
| *Human IL-8* | Forward | CTG GCC GTG GCT CTC TTG |
|  | Reverse | CCT TGG CAA AAC TGC ACC TT |
| *Human BNIP3* | Forward | GCC ATC GGA TTG GGG ATC TAT |
|  | Reverse | GCC ACC CCA GGA TCT AAC AG |
| *Human P16* | Forward | GTG GAC CTG GCT GAG GAG |
|  | Reverse | CTT TCA ATC GGG GAT GTC TG |
| *Human P53* | Forward | GGC CCA CTT CAC CGT ACT AA |
|  | Reverse | GTG GTT TCA AGG CCA GAT GT |
| *Human CD55* | Forward | CAG CAC CAC CAC AAA TTG AC |
|  | Reverse | CTG AAC TGT TGG TGG GAC CT |
| *Human CXCR4* | Forward | TAC ACC GAG GAA ATG GGC TCA |
|  | Reverse | AGA TGA TGG AGT AGA TGG TGG G |
| *Human VIMENTIN* | Forward | AGG CAA AGC AGG AGT CCA CTG A |
|  | Reverse | ATC TGG CGT TCC AGG GAC TCA T |
| *Human TGF-β1* | Forward | CCC AGC ATC TGC AAA GCT C |
|  | Reverse | GTC AAT GTA CAG CTG CCG CA |
| *Human TNF-α* | Forward | TCT TCT CGA ACC CCG AGT GA |
|  | Reverse | CCT CTG ATG GCA CCA CCA G |
| *Human IL-6* | Forward | GCA CTG GCA GAA AAC AAC CT |
|  | Reverse | TCA AAC TCC AAA AGA CCA GTG A |
| *Human HGF* | Forward | GAT GGC CAG CCG AGG C |
|  | Reverse | TCA GCC CAT GTT TTA ATT GCA |
| *Human PECAM1* | Forward | AAG TGG AGT CCA GCC GCA TAT C |
|  | Reverse | ATG GAG CAG GAC AGG TTC AGT C |
| *Human MCP-1* | Forward | AGA ATC ACC AGC AGC AAG TGT CC |
|  | Reverse | TCC TGA ACC CAC TTC TGC TTG G |
| *Human ADAM10* | Forward | TCC CCT TGC AAC GAT TTT AG |
|  | Reverse | AAT ACT GCC CAC CAA TGA GC |
| *Human EGLN1* | Forward | TTT TTC TGG TCT GAC CGT CGC A |
|  | Reverse | CCC TCA CAC CTT TTT CAC CTT GT |
| *Human HES1* | Forward | CCT ATT ATG GAG AAA AGA C |
|  | Reverse | GAG GTG CTT CAC TGT CAT T |
| *Human FGF2* | Forward | AGC GGC TGT ACT GCA AAA AC |
|  | Reverse | GTA GCT TGA TGT GAG GGT CG |
| *Human MMP9* | Forward | CCA CTG CTG GCC CTT CTA CG |
|  | Reverse | CGA TGG CGT CGA AGA TGT TCA C |
| *Human IL-12B* | Forward | AAC TTG CAG CTG AAG CCA TT |
|  | Reverse | GAC CTG AAC GCA GAA TGT CA |
| *Human IL-4* | Forward | ACT TTG AAC AGC CTC ACA GAG |
|  | Reverse | TTG GAG GCA GCA AAG ATG TC |
| *Human Fibronectin* | Forward | TGA AAG ACC AGC AGA GGC ATA AG |
|  | Reverse | CTC ATC TCC AAC GGC ATA ATG G |
| *Rat Gapdh* | Forward | AAC GAC CCC TTC ATT GAC CTC |
|  | Reverse | CCT TGA CTG TGC CGT TGA ACT |
| *Rat Bax* | Forward | GAG GCA GCG GCA GTG ATG |
|  | Reverse | GAT CCT GGA TGA AAC CCT GTA GC |
| *Rat Timp-2* | Forward | CCA GAA GAA GAG CCT AAA CCA |
|  | Reverse | GTC CAT CCA GAG GCA CTC ATC |


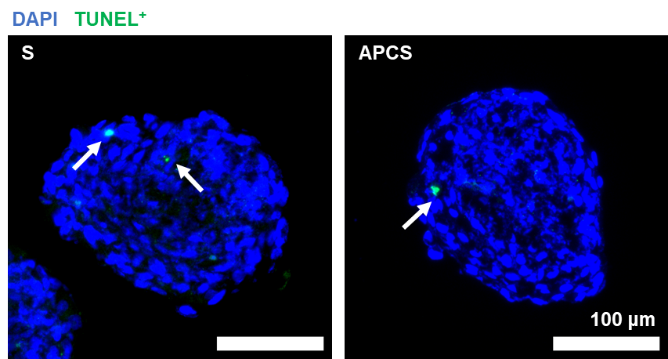


**Figure S1.** **Representative TUNEL staining images of spheroids.** (TUNEL^+^: green and nuclei: blue, scale bar: 100 μm).


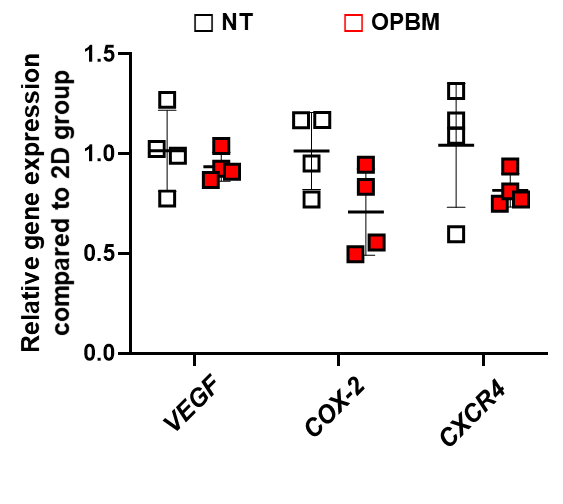


**Figure S2. Relative expression of angiogenic factors in hADSCs.** (A) Relative expression of *VEGF*, *COX-2*, and *CXCR4* in hADSCs 48 h after preconditioning using red light. The NT group was used as the control (n = 4).


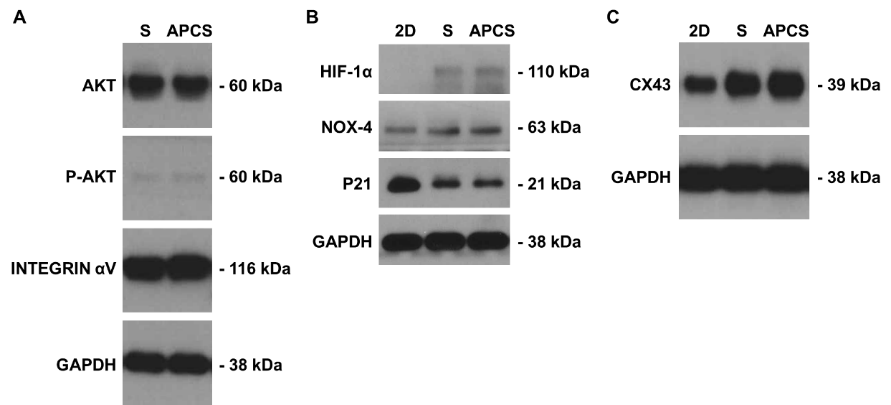


**Figure S3.** (A) Uncropped blot corresponding to Figure 1E. (B) Uncropped blot corresponding to Figure 2D. (C) Uncropped blot corresponding to Figure 2H.
